# Application of apigeninidin‐rich red sorghum biocolorant in a fermented food improves product quality

**DOI:** 10.1002/jsfa.9427

**Published:** 2018-12-04

**Authors:** Folachodé UG Akogou, Tessa S Canoy, Adéchola PP Kayodé, Heidy MW den Besten, Anita R Linnemann, Vincenzo Fogliano

**Affiliations:** ^1^ Laboratory of Valorization and Quality Management of Food Bio‐Ingredients (LaBio), Faculté des Sciences Agronomiques Université d'Abomey‐Calavi Cotonou Benin; ^2^ Food Quality and Design Department of Agrotechnology and Food Sciences, Wageningen University & Research Wageningen The Netherlands; ^3^ Laboratory of Food Microbiology Department of Agrotechnology and Food Sciences, Wageningen University & Research Wageningen The Netherlands

**Keywords:** apigeninidin, maize dough, fermentation, antioxidant activity, nutritional quality, volatile compounds

## Abstract

**BACKGROUND:**

The ‘clean label’ trend is pushing the food industry to replace synthetic colorants with plant‐based colorants. However, technological efficacy and undesirable side effects restrict the use of plant‐based colorants in industrial applications. This research studied the production of fermented maize dough coloured by apigeninidin‐rich red sorghum biocolorant, as practised for centuries in West Africa, as a model to assess the impact of the biocolorant on nutritional and sensorial quality of foods.

**RESULTS:**

A 3‐day fermentation of a dyed maize dough (containing 327 µg g^−1^ dry matter of apigeninidin) by *Pichia kudriavzevii* and *Lactobacillus fermentum* led to a degradation of 69% of the apigeninidin content, causing a clearly visible colour difference (ΔE*_00_ 17.4). The antioxidant activity of fermented dyed dough (DD) increased by 51% compared to fermented non‐dyed dough (NDD). However, the phytate dephosphorylation and volatile organic compound concentrations were lower in DD than in NDD. This suggests a lower mineral solubility and change in the sensory quality of fermented DD.

**CONCLUSION:**

Apigeninidin extract from sorghum leaf sheaths proved to be a bioactive red biocolorant with potential in fermented foods. The formation of new antioxidant compounds needs further investigation, as does the impact on the development of volatile compounds. © 2018 The Authors. *Journal of The Science of Food and Agriculture* published by John Wiley & Sons Ltd on behalf of Society of Chemical Industry.

## INTRODUCTION

Colour has been added to food for centuries.^1^ Synthetic and natural colorants share 40% and 31% of the colorant market, respectively.^2^ However, the demand for natural colorants is predicted to increase by an annual rate of 5–10%.^1^ This increasing interest for natural colorants is supported by (i) legislation in favour of replacement of synthetic colorants because of increasing concerns about their safety and (ii) consumer awareness of the safety risks associated with synthetic colorants.^2^ The anthocyanins represent the most diverse group of plant‐based colorants, with more than 600 molecular structures identified to date.^3^ In addition to their colouring properties, anthocyanins are of interest to the food industry because of their health‐supporting properties (i.e. their activity as antioxidants).^3^ However, their low stability to processing conditions (e.g. conditions of mild acid and neutral pH, high temperature, light and oxygen) generally limits their use as food colorants.^4^

In West Africa, breakfast usually consists of fermented, cereal‐based porridges that are prepared from maize or sorghum sourdough.^5^ Fermentation of the sourdough involves lactic acid bacteria such as *Lactobacillus fermentum* and yeasts, including *Saccharomyces cerevisiae* and *Pichia kudriavzevii*.^5^ Sourdough fermentation increases the amounts of fermentable carbohydrates, amino acids, small peptides and soluble minerals. Furthermore, several groups of volatile organic compounds (VOCs, i.e. hydrocarbons, esters, carbonyls, alcohols and acids) are produced by the microbial conversion of amino acids and polyphenols, providing a characteristic contribution to the sensory profile of the final product.^5^, ^6^

Despite these positive aspects, the nutritional profile of cereal‐based products is far from complete and the combination with ingredients rich in proteins, essential fats and/or phytochemicals is common practice.^5^, ^7^ In West Africa, a red sour maize porridge, obtained by incorporation of a red extract from dye sorghum leaf sheaths, is produced and consumed by both infants and adults.^8^ Red cereal porridges are preferred by consumers in Benin because of their sensorial and alleged nutritional properties.^9^ The dye sorghum extract contains apigeninidin, which is a 3‐deoxyanthocyanidin with potential application in the food industry because of its stability to pH, heat treatment and bleaching.^10^, ^11^ In addition, the antioxidant activity of the apigeninidin‐rich extract suggests health benefits for consumers.^12^ Bai *et al*.^13^ and Izquierdo‐Cañas *et al*.^14^ suggested that the fermentation of an anthocyanin‐rich food matrix with only lactic acid bacteria (e.g. *L. fermentum*) leads to (i) decarboxylation of phenolic acids and (ii) conversion of anthocyanins into pyranoanthocyanins and vinylphenol adducts. The use of yeasts (e.g. *P. kudriavzevii*) is recommended for efficient anthocyanin degradation.^15^ The Beninese sourdough process, involving both yeast (e.g. *P. kudriavzevii*) and lactic acid bacteria (*L. fermentum*), provides a food model suitable to investigate the stability of apigeninidin and its potential added value to fermented starchy foods. The apigeninidin‐rich extract application in dough fermentation according to practices in Benin and its effect on the nutritional and sensorial properties of the food product are not well documented. Therefore, this study investigated apigeninidin stability, antioxidant activity, sugar content, phytate (i.e. inositol hexaphosphate (IP6)), oxalate, phosphate and VOCs during fermentation of maize dough enriched with red apigeninidin‐rich extract from sorghum leaf sheaths as a contribution to the potential use of this biocolorant in industrial applications.

## MATERIAL AND METHODS

### Maize processing

White maize grains were bought from the local market of Abomey‐Calavi, Benin. The maize grains were cleaned to remove stones, washed in demineralised water and dried in an oven (Venticell 55, MMM Medcenter Einrichtungen, Planegg, Germany) at 60 °C for 24 h. A mill (RotorMill Pulvrisette‐14) equipped with a 0.2 mm sieve was used to mill the grain into flour.

### Extraction of biocolorant from sorghum leaf sheaths

The alkaline extraction method reported by Akogou *et al*.^8^ was used to obtain a sorghum watery extract (SWE). Briefly, dry sorghum leaf sheaths (11.1 g) were suspended in demineralised water (1000 mL) alkalinised by *kanwu* (1.5 g), an alkaline rock salt. The mixture was stirred for 20 min at room temperature to obtain SWE. The liquid and solid particles were separated using a 0.3 mm sieve. The SWE was centrifuged at 3000 rpm at 4 °C for 30 min to obtain the supernatant. The control was a solution of *kanwu* (1.5 g L^−1^), the pH of which was adjusted to 8.7 with citric acid (1 mol L^−1^) to match the pH of the SWE. A 0.2 µm AcroVac filter unit (VWR, Netherlands) was used to filter‐sterilise both the SWE and the control solution of *kanwu*.

### Inoculum preparation

Lactobacilli (i.e. *L. fermentum*) and yeast (i.e. *Candida krusei*, also known as *P. kudriavzevii*) with the highest distribution frequency during maize dough fermentation were selected to prepare the incocula.^16^, ^17^ The strains of *P. kudriavzevii* and *L. fermentum* were provided by the Laboratory of Food Microbiology, Wageningen University, Netherlands, and isolated from fermented *Ziziphus mauritiana* fruit and soybean soak water, respectively. One loopful of *P. kudriavzevii* and *L. fermentum* kept at −80 °C were inoculated on malt extract (ME) and Man Rogosa Sharpe (MRS) agar plates, respectively. The inoculated ME and MRS agar plates were incubated at 30 °C in an incubator (VENTI‐Line VL 115, VWR) under (i) aerobic conditions for 24 h and (ii) microaerophilic conditions for 48 h, respectively. One colony each of *P. kudriavzevii* and *L. fermentum* was transferred to 9 mL of ME broth and MRS broth, respectively. The tubes were incubated at 37 °C for 18 h. Next, concentrations of the inocula were determined by plate counting, resulting in 6.8 and 8.6 log cfu mL^−1^ for *P. kudriavzevii* and *L. fermentum*, respectively.

### Inoculation and fermentation

The DD containing 6 and 4 log cfu g^−1^ of *L. fermentum* and *P. kudriavzevii*, respectively, was prepared by mixing 112 mL sterile sorghum biocolorant, 0.56 mL *L. fermentum* inoculum, 0.36 mL of *P. kudriavzevii* inoculum and 112 g of maize flour. The same procedure was used to prepare the NDD, except that the sterile sorghum biocolorant was replaced with the sterile control solution of *kanwu*. For pictures of DD and NDD, see supporting information. The initial concentrations of *L. fermentum* and *P. kudriavzevii* in maize dough corresponded to the initial concentration of lactobacilli and yeast in homemade maize dough in Benin.^18^ The samples of DD and NDD were transferred to sterile boxes. Five samples of DD and NDD were prepared for sampling at 0, 5, 24, 48 and 72 h of fermentation, and placed at 30 °C in an incubator (VENTI‐Line VL 115, VWR). The pH, colour and VOCs were measured on the fresh DD and NDD at each time point. Contents of apigeninidin, sugar, phytate, oxalate and phosphate, as well as antioxidant activity, were measured on freeze‐dried DD and NDD. All experiments (including inoculum preparation, inoculation and fermentation) were performed in duplicate.

## ANALYSES

### pH and colour

The pH values of 30 g fresh DD and NDD were measured with a pH meter (pHenomenal pH 1100 L,VWR International), calibrated with two buffers of pH 7 and pH 4 (Merck, Darmstadt, Germany). Colour (L*, a*, b*) of the fresh DD and NDD was measured with a spectrocolorimeter (ColorFlex, HunterLab, Reston, VA, USA; illuminant D65). The L*, a* and b* values were used to calculate chroma (C*) and hue (*h*°). In addition, the total colour difference (ΔE*_00_) of DD and NDD was calculated using the time zero colour as reference for DD and NDD, respectively.^19^

### Phenolics and apigeninidin

Phenolic compounds were extracted according to the method of Kayodé *et al*.^12^ Freeze‐dried samples (100 mg) were added to HCl–methanol (1% v/v) (3 mL) and stirred for 1 h at room temperature, followed by centrifugation at 5000 × *g* for 10 min and collection of the supernatant. The pellet was extracted again under the same conditions. Apigeninidin was measured in the resulting 6 mL extract. Before high‐performance liquid chromatography (HPLC) injection, each extract was mixed with an equal volume of formic acid (10%) in milli‐Q water and filtered with 0.2 µm RC filters. Apigeninidin was measured with an Ultimate 3000 RS HPLC system, equipped with a diode array detector (DAD‐3000 RS; Thermo Scientific Dionex, Waltham, MA, USA), a quaternary pump (LPG‐ 3000 RS; Thermo Scientific Dionex) and a Polaris C18‐A column (150 × 4.6 mm, Varian, Palo Alto, CA, USA). Formic acid (10%) in milli‐Q water (A) and methanol (100%) (B) were used as eluents. The flow rate was 1 mL min^−1^, with a total elution programme of 35 min, as follows: 0–20 min from 5% B to 60% B; 20–25 min from 60% B to 100% B; 25–30 min 100% B; 30–31 min from 100% B to 5% B; 31–35 min 5% B. UV spectra were recorded from 220 to 700 nm. Apigeninidin (Extrasynthese, Genay, France) was used as standard for identification and quantification at 480 nm.

### Sugars

Freeze‐dried DD and NDD samples (100 mg) were added to ethanol–milli‐Q water (50:50, v/v) (40 mL) and vortexed for 1 min. After incubation at 50 °C for 1 h in a water bath, the mixture was centrifuged for 10 min at 3000 rpm and the supernatant was collected. The supernatant was filtered with a 0.2 RC filter before identification and quantification of sugars using HPLC with d‐glucose (Merck) as standard. The HLPC system was equipped with an evaporative light scattering (ELS) detector (PL‐ELS 2100; Polymer Laboratories, Church Stretton, UK), a pump and a Grace Prevail carbohydrate ES 5 µm, 250 × 4.6 mm column (Grace, Columbia, MD, USA). The elution programme was isocratic with acetonitrile (A) (75%) and milli‐Q water (B) (25%) and with a total running time of 30 min. The flow rate was 0.8 mL min^−1^.

### Phytate, phosphate and oxalate

Concentrations of phytate, phosphate and oxalate were analysed by HPLC with conductivity detection according to Bentsink *et al*.,^20^ with some modifications. To 20 mg freeze‐dried DD and NDD, 1 mL of 0.5 mol L^−1^ HCl containing 50 mg L^−1^ *trans*‐aconitate (Fluka, Buchs, Switzerland) was added. The mixture was extracted at 100 °C for 15 min. After centrifugation at 13 300 rpm for 5 min, the supernatant was collected and diluted five times with milli‐Q water. The diluted supernatant was injected into a Dionex ICS2500 system with an AS11‐HC column and AG11‐HC guard column, set at a flow time of 5 mL min^−1^. The following eluent and elution times were used: 25–100 mmol L^–1^ NaOH from 0 to 15 min; 500 mmol L^–1^ NaOH from 15 to 20 min to rinse the column, and 25 mmol L^–1^ NaOH from 20 to 35 min to equilibrate the column. Data were collected from the HPLC instrument as amounts of anions in mg L^−1^. *trans*‐Aconitic acid (Fulka), citric acid monohydrate (BDH), sodium phosphate dibasic (Merck) and phytic acid sodium salt hydrate (Sigma, Darmstadt, Germany) were used as standards for identification and quantification.

### Antioxidant activity

The QUENCHER method was used to determine the total antioxidant activity of the solid material without extraction.^21^ A stock solution of 2,2‐diphenyl‐1‐picrylhydrazyl (DPPH) was prepared by dissolving 10 mg DPPH (Sigma) in 250 mL ethanol (Merck) (50%). The solution was incubated for 23 h and its absorbance at 525 nm was 0.5–0.8 before use. Freeze‐dried samples of DD and NDD were diluted with cellulose at a ratio of 1:1. In 5 mL Eppendorf tubes, 5, 10, 20 and 40 mg of the diluted freeze‐dried DD and NDD were prepared.

Trolox (Sigma Aldrich, Zwijndrecht, Netherlands) was used as standard. Standard solutions of 4, 10, 20 and 30 mmol L^–1^ Trolox in ethanol (100%) were prepared. The tubes for the calibration curve were prepared by adding 10 µL of 4, 10, 20 and 30 mmol L^–1^ Trolox to 10 mg cellulose. The blank was prepared by adding 10 µL ethanol (100%) to 10 mg cellulose. Four tubes of blank were prepared.

A volume of 5 mL DPPH solution was added to the tubes containing samples, calibration points and blank. The tubes were incubated for 100 min at room temperature and stirred with a shaker (Heidolph Multi Reax, Germany). Subsequently, the tubes were centrifuged for 5 min at 9000 × *g* and the absorbance was measured at 525 nm with a UV–visible spectrophotometer (Cary WinUV 50, Varian). The antioxidant activity in freeze‐dried DD and NDD was expressed in mmol g^−1^ Trolox equivalent using Eqn (1):

(1) $$\text{Antioxidant activity}\;\left(\text{mmol}\;g^{-1}\right)=\frac{{\text{Trolox}}_{50}\;\left(\text{mmol}\right)}{{\text{Sample}}_{50}\;\left(\mathrm{mg}\right)}\times 10^{3}$$

where Trolox_50_ is the concentration of Trolox (mmol) at which the initial blank absorbance is reduced by half, Sample_50_ is the amount of sample (mg) at which the initial blank absorbance is reduced by half, and 10^3^ is the conversion factor from mmol mg^−1^ to mmol g^−1^.

### Volatile compounds

VOCs of DD and NDD at 0 and 72 h of fermentation were determined by headspace measurements. DD and NDD samples (0.5 g) were transferred to 500 mL bottles. The volatile profile was analysed with a PTR‐ToF‐MS 8000 instrument (Ionicon Analytik GmbH, Innsbruck, Austria) with the following instrumental conditions for the proton transfer reaction: drift voltage 999.0 V, drift temperature 59.9 °C and pressure drift 3.8 mbar, affording an *E*/*N* value of 134 Td. Sampling was performed with a flow of 52 mL min^−1^. The mass (*m*/*z*) was recorded from 20 to 300. The acquisition rate was 1 spectrum s^−1^. A total run of 60 s was applied to each sample as follows: 0–10 s to collect the spectrum of the room and 10–60 s to collect the spectrum of the samples by connecting the bottle containing the samples to the inlet. The dead time correction, internal calibration of the mass spectra, peak extraction and calculations of the VOCs from the peaks were according to Cappellin *et al*.^22^ and Lindinger and Jordan.^23^ The concentrations are reported in ppbv (parts per billion by volume). The averages of the cycles 0–10 and 40–50 were calculated and used as the VOCs of the blank and samples, respectively. For each sample, the blank was subtracted.

## DATA ANALYSIS

Data are expressed as mean ± standard deviation. Data were analysed with SPSS Statistical software version 23. One‐way analysis of variance (ANOVA) with Tukey's HSD post hoc was used for normally distributed data with homogeneity of variance (i.e. for phosphate, phytate and citrate). When the conditions for the application of ANOVA were not fulfilled (i.e. for pH, L*, C*, *h*°, apigeninidin, oxalate, antioxidant activity and sugars), the non‐parametric, Kruskal–Wallis and Mann–Whitney pair‐wise comparisons were used. Analysis of variance and principal component analysis (PCA) were performed to select the masses that explain the differences in the aroma profiles of the DD and the NDD before and after fermentation.

## RESULTS AND DISCUSSION

**Figure 1:**
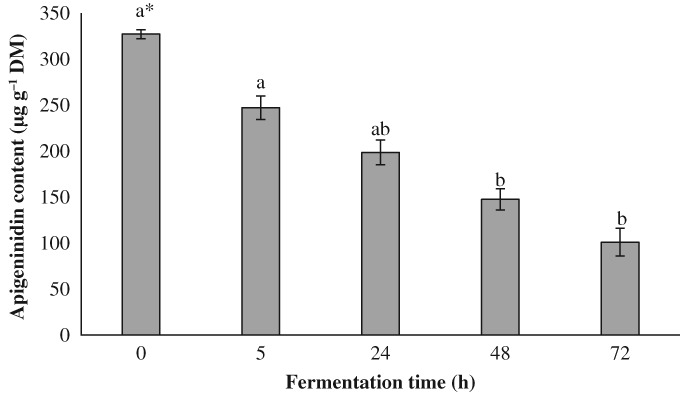
Apigeninidin content during fermentation of dyed maize dough.

Figure 1 presents the apigeninidin content of DD during fermentation. After 3 days of fermentation, 69% of the apigeninidin content was degraded. Apigeninidin is usually described as stable to most common processing conditions (pH, temperature and bleaching).^10^, ^11^ Therefore, the pH drop cannot explain the extensive decrease in apigeninidin observed during dough fermentation in the present study. The action of microorganisms (*L. fermentum* and *P. kudriavzevii*) used as inoculum apparently affected the apigeninidin content.^24^ Indeed, yeast and lactic acid bacteria can affect the anthocyanin concentration. Anthocyanin could be absorbed by yeast, based on the hydrophobic interaction between the anthocyanins and the yeast cell walls.^25^ The less hydroxylated anthocyanins, such as the 3‐deoxyanthocyanidins from sorghum (e.g. apigeninidin), could then be absorbed and degraded. In addition, the enzymatic activity of lactic acid bacteria (e.g. decarboxylase and reductase) on anthocyanins could have contributed to the degradation of apigeninidin into phenolic acids and VOCs.^24^, ^26^

Table 1 presents the total colour difference (ΔE*_00_), lightness (L*), chroma (C*) and hue (*h*°) of the DD and NDD during fermentation. The ΔE*_00_ of DD and NDD increased during fermentation. ΔE*_00_ is commonly classified as small (when ΔE*_00_ < 1.5), distinct (1.5 < ΔE*_00_ < 3) or large (ΔE*_00_ > 3).^27^ From 0 to 24 h of fermentation, ΔE*_00_ of NDD was small, but >3 at 48 and 72 h of fermentation. Indeed, the difference in colour of the NDD could visually be perceived from 48 h of fermentation onwards. Conversely, ΔE*_00_ of DD was >3 already after 5 h of fermentation and increased to 17.4 at 72 h of fermentation. L* values increased during fermentation of NDD, whereas C* and *h*° values remained stable. Conversely, L* and *h*° values of DD increased during fermentation, whereas C* values remained stable. From the above data, the colour change of DD and NDD after fermentation can be described as a visible change from red to orange‐red and from yellow to light yellow, respectively. The decrease in apigeninidin content and production of organic acids (e.g. lactic acid) affecting pH and colour intensity both contributed to the loss of total colour density during fermentation.^28^

**Table 1:** Colour of dyed and non‐dyed maize dough during fermentation

Samples	Time (h)	ΔE*_00_	L*	C*	*h*°
Non‐dyed dough	0	0 ± 0	75.0 ± 0.7b	15.4 ± 0.6b	86.9 ± 0.2c
	5	0.8 ± 0.6	74.6 ± 0.4b	14.8 ± 0.5b	87.9 ± 0.0c
	24	0.9 ± 0.4	74.2 ± 0.0b	15.0 ± 0.0b	88.8 ± 0.1c
	48	4.5 ± 1.1	81.3 ± 0.9a	15.3 ± 0.4b	87.9 ± 0.5c
	72	5.3 ± 0.4	82.5 ± 0.2a	16.1 ± 0.0b	88.0 ± 0.1c
Dyed dough	0	0 ± 0	44.0 ± 1.0d	35.3 ± 0.2a	32.5 ± 0.2a
	5	5.0 ± 2.0	48.4 ± 1.2d	32.6 ± 0.8a	37.7 ± 0.6a
	24	7.9 ± 0.8	49.8 ± 0.2d	34.9 ± 0.3a	45.0 ± 0.3b
	48	15.1 ± 0.1	57.8 ± 0.9c	36.7 ± 0.5a	46.3 ± 0.6b
	72	17.1 ± 1.5	59.7 ± 0.5c	35.4 ± 0.3a	48.4 ± 0.4b

Mean ± standard deviation; total colour difference compared to colour at 0 h: small (ΔE*_00_ < 1.5), distinct (1.5 < ΔE*_00_ < 3) or large (ΔE*_00_ > 3); values in a column with the same letter are not significantly different at 5%.

**Figure 2:**
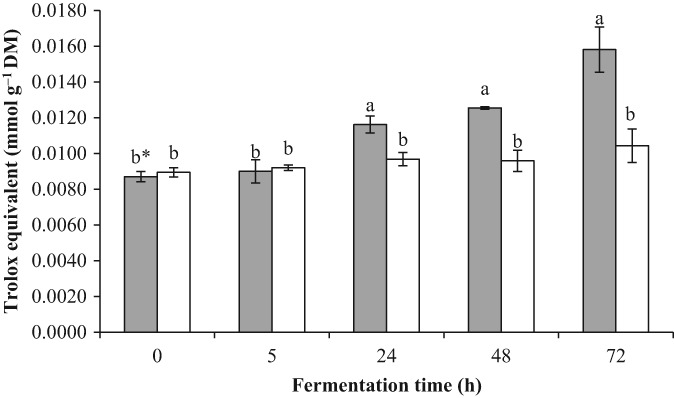
Antioxidant activity of dyed (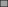) and non‐dyed (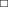) maize dough during fermentation.

Figure 2 shows the antioxidant activity during fermentation. Fermentation of DD from 24 to 72 h increased the antioxidant activity by 20% to 51%, respectively, compared to NDD. In cereal foods, up to 70% of the antioxidant components are insoluble and cannot be measured in a solvent extract.^21^ The QUENCHER method allows a direct antioxidant measurement of the food matrix and provides more accurate data on the antioxidant activity of a food matrix.^21^ Clearly, the incorporation of apigeninidin‐rich sorghum extract in maize dough followed by fermentation improved this functional product quality aspect. Apigeninidin is reported to possess significant antioxidant activity.^12^ However, no difference in antioxidant activity was observed between DD and NDD at time zero or during the first 5 h of fermentation. Boveris *et al*.^29^ suggested a minimum dose–response of 200 µg mL^−1^ of apigeninidin for scavenger activity towards hydroxyl radicals, quinones and/or nitric oxide. The application of apigeninidin at a concentration up to 327 µg g^−1^ DM in dough might be below the minimum dose–response for scavenger activity in solid foods (e.g. dough). Furthermore, the decrease in apigeninidin content and simultaneous increase in antioxidant activity after a minimum fermentation of 24 h suggest the formation of new antioxidant compounds.

Table 2 presents the changes in pH, sugar content, mineral chelating compounds (phytate and oxalate) and phosphate content in DD and NDD during fermentation. The highest pH drop and sugar increase were recorded between 5 and 24 h of fermentation in both doughs.^30^, ^31^ The oxalate content remained stable in both doughs. Conversely, phytate content (IP6) was higher in DD (8.4 mg g^−1^) than in NDD (7.5 mg g^−1^) at 0 h.^30^ This suggests that the application of sorghum biocolorant increased the phytate content of the dough. Nevertheless, fermentation efficiently lowered the phytate content from 7.5–8.4 to 6.1–6.3 mg g^−1^ in both doughs after 72 h of fermentation. The phosphate content increased accordingly from 0.3–0.4 to 0.9 mg g^−1^ after 72 h of fermentation as a result of phytate degradation into its lower polymers. Furthermore, the Pearson correlation (*r* ^2^ = 0.87) between phytate degradation and phosphate formation during fermentation was significant at 0.01 level. Phytate and oxalate are common mineral chelating compounds of importance in cereals and affect the solubility of essential minerals in the human gut.^30^, ^31^ However, the complexation of oxalate with cations reduces the solubility of oxalate in the gut and is therefore considered as a strategy to reduce the absorption of oxalate, thereby preventing the formation of kidney stones.^31^ The decrease in molar concentration of phytate and increase in molar concentration of phosphate were 0.003 and 0.006 mmol g^−1^, respectively, in DD and 0.002 and 0.007 mmol g^−1^, respectively, in NDD. For a one unit decrease in phosphorylation level, the molar concentration of degraded phytate should be equal to the molar concentration of formed phosphate. A higher ratio of molar concentration of formed phosphate to the molar concentration of degraded phytate suggests a higher level of dephosphorylation. Ratios of the molar concentration of formed phosphate to the molar concentration of degraded phytate were 2 and 3.5 in DD and NDD, respectively. A minimum phytate dephosphorylation level of 2 (from IP6 to inositol tetraphosphate (IP4)) could increase mineral solubility.^30^ However, the mineral solubility in DD might be lower than in NDD. Phytate dephosphorylation would increase the cation availability, which in turn could bind oxalate, reducing its solubility.^30^, ^31^ However, the lower dephosphorylation of phytate in DD suggested (i) less soluble minerals and (ii) more soluble oxalate, increasing the risk of kidney stones in comparison to NDD.

**Table 2:** Physicochemical characteristics of dyed and non‐dyed maize dough during fermentation

Samples	Time (h)	pH	Sugars (mg g^−1^ DM)	Phytate (mg g^−1^ DM)	Phosphate (mg g^−1^ DM)	Oxalate (mg g^−1^ DM)
Non‐dyed dough	0	6.3 ± 0.1a	nd	7.5 ± 0.2b	0.3 ± 0.01e	0.3 ± 0.02a
	5	6.3 ± 0.1a	nd	7.6 ± 0.4b	0.5 ± 0.03d	0.3 ± 0.01a
	24	4.3 ± 0.1b	58.7 ± 0.7a	6.7 ± 0.5cd	0.7 ± 0.01c	0.3 ± 0.01a
	48	4.3 ± 0.0b	61.7 ± 1.5a	6.5 ± 0.1cd	0.8 ± 0.00a	0.3 ± 0.00a
	72	4.3 ± 0.0b	50.4 ± 2.4b	6.3 ± 0.4d	0.9 ± 0.00a	0.4 ± 0.00a
Dyed dough	0	6.3 ± 0.1a	nd	8.4 ± 0.5a	0.4 ± 0.01e	0.3 ± 0.00a
	5	6.3 ± 0.1a	nd	7.8 ± 0.2ab	0.4 ± 0.03d	0.3 ± 0.01a
	24	4.3 ± 0.1b	57.1 ± 1.0a	7.2 ± 0.1b	0.6 ± 0.00c	0.3 ± 0.01a
	48	4.2 ± 0.1b	58.3 ± 2.0a	6.2 ± 0.1d	0.73 ± 0.01b	0.3 ± 0.00a
	72	4.1 ± 0.0b	53.4 ± 2.2b	6.1 ± 0.01d	0.9 ± 0.03a	0.3 ± 0.01a

nd, not detected.

Mean ± standard deviation; values in a column with the same letter are not significantly different at 5%.

**Figure 3:**
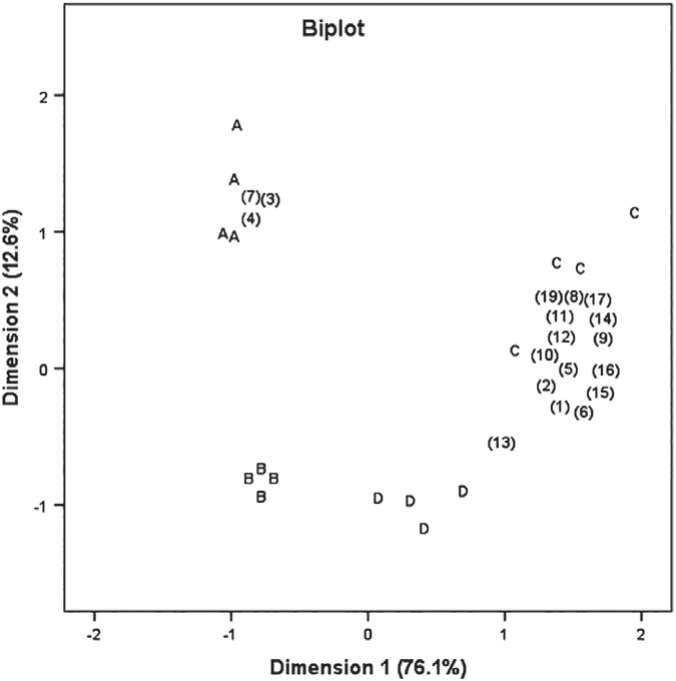
Normalised biplot clustering the volatile organic compounds in the doughs: A, dyed dough; B, non‐dyed dough; C, fermented non‐dyed dough; D, fermented dyed dough. (1) *m*/*z* 29.039; (2) *m*/*z* 31.017; (3) *m*/*z* 33.036; (4) *m*/*z* 37.092; (5) *m*/*z* 43.019; (6) *m*/*z* 47.05; (7) *m*/*z* 51.044; (8) *m*/*z* 71.048; (9) *m*/*z* 89.06; (10) *m*/*z* 107.076; (11) *m*/*z* 117.091; (12) *m*/*z* 117.132; (13) *m*/*z* 121.07; (14) *m*/*z* 135.106; (15) *m*/*z* 135.147; (16) *m*/*z* 136.138; (17) *m*/*z* 163.136; (18) *m*/*z* 177.139; (19) *m*/*z* 225.267.

Figure 3 presents the clustering of the VOCs in the doughs as determined by PTR‐MS at 0 and 72 h of fermentation. The two first dimensions of the PCA biplot accounted for 88.7% of the total variation. The application of sorghum biocolorant changed the VOCs in DD and can be related to three masses (*m*/*z* 33.036, 37.092 and 51.044). Furthermore, the masses related to fermented NDD were not related to fermented DD. This demonstrates differences in the development of VOCs during fermentation of DD and NDD.

Table 3 presents the profile of VOCs contributing to the differences between the doughs, their tentative identification by exact mass and their concentration (in ppbv). Five groups of compounds (i.e. alkenes, alcohols, esters, aldehydes and acids) were tentatively identified. The application of sorghum biocolorant appears to increase the concentration of aldehyde (methyl aldehyde), alcohol (methanol) and esters in DD at 0 h of fermentation. The reaction of methyl radicals with the alkaline solvent could lead to the formation of methanol in sorghum biocolorant and subsequently to methyl aldehyde and esters.^36^ However, the fermentation of DD appears to lower the amounts of alkene (ethylene), alcohol (ethanol), aldehyde (butenal), acid (hexanoic acid) and esters (methyl propanoate). Anthocyanins are able to affect the activity of microbiological enzymes (e.g. carbohydrate‐utilising enzymes) and may contribute to the differences in VOCs between fermented DD and fermented NDD.^37^, ^38^ A difference in the food substrate (such as the concentration of anthocyanidins) could generate stress‐induced damage in microorganisms and therefore require a high amount of energy (e.g. ATP) from the microorganisms.^38^ Without an increase in ATP, the intracellular metabolism of the microorganisms (e.g. involved in the formation of the VOCs) could be lower.^38^ Adaptation of the intracellular metabolism by anthocyanins might be possible after a 3‐day transition and under a continuous supplementation of anthocyanins.^38^ However, the fermentation time was too short for microorganisms to complete their adaptation; hence insufficient energy is presumably the main factor leading to a lower amount of VOCs (alcohol, aldehyde, acids and esters).

**Table 3:** Tentatively identified volatile organic compounds (ppbv) of dyed and non‐dyed maize dough as affected by fermentation

*m*/*z* measured	Formula	Tentative identification	Non‐ fermented		Fermented (72 h)	
			Non‐dyed dough	Dyed dough	Non‐dyed dough	Dyed dough
29.039	C_2_H_5_^+^	Ethylene	264.3 ± 28.8c	574.9 ± 168.2c	6263.9 ± 202.4a	5468.8 ± 382.9b
31.017	CH_3_O^+^	Methyl aldehyde	21.1 ± 7.3c	259.5 ± 109.2b	1692.1 ± 222.3a	1574.7 ± 294.7a
33.036	CH_5_O^+^	Methanol^32^	24 702.7 ± 3495.2b	218 209.6 ± 54 144a	16.0 ± 28.7c	58.6 ± 51.0c
37.092	n.a.	n.i.	2195.4 ± 306.9b	73 305.1 ± 11 531.7a	2.2 ± 2.5c	0.8 ± 1.7c
43.019	C_2_H_3_O^+^	Fragment/ester^33^	55.7 ± 27.5d	983.6 ± 203.1c	74 743.2 ± 12 411.5a	41 754.26 ± 10 602.4b
47.05	C_2_H_7_O^+^	Ethanol^34^	2091.5 ± 662.4c	18 232.6 ± 9386c	518 851.6 ± 33 636.6a	430 576.3 ± 48 259.5b
51.044	n.a	n.i.	1515.4 ± 265.3b	13 084.1 ± 3764a	5.9 ± 1.3c	10.1 ± 2.1b
71.048	C_4_H_7_O^+^	Butenal/ester^32^, ^33^	1.8 ± 0.5c	42 ± 37.1c	8976.9 ± 3719.7a	2623.2 ± 797.7b
89.060	C_4_H_9_O_2_^+^	Methyl propanoate^35^	2.4 ± 4.1c	114.6 ± 86.7c	376 885 ± 95 119.2a	190 002.1 ± 42 048.1b
107.076	n.a	n.i.	1.1 ± 0.5c	27.7 ± 14.3c	5498.0 ± 1536.9a	2682.6 ± 472.8b
117.091	C_6_H_13_O_2_^+^	Hexanoic acid/ester^34^	0 ± 0c	10.5 ± 5.5c	423 179 ± 143 625.4a	123 807.8 ± 40 128.1b
117.132	n.a	n.i.	0 ± 0c	13.5 ± 6.9c	131 458.2 ± 51 278.1a	44 387.3 ± 19 957.3b
121.07	C_8_H_9_O^+^	Methylbenzaldehyde–coumaran^32^	0.1 ± 0.1c	4.8 ± 1.9c	115.0 ± 40.1b	223.3 ± 84.0a
135.106	C_6_H_15_O_3_^+^	n.i.	0.2 ± 0.2d	7.8 ± 1.9c	29 082.0 ± 8806.0a	11 155.9 ± 3471.7b
135.147	n.a	n.i.	0.4 ± 0.4d	12.1 ± 3.7c	16 261.4 ± 3439.7a	10 657.4 ± 3384.0b
136.138	n.a	n.i.	0.0 ± 0.0d	0.6 ± 0.2c	1331.5 ± 331.8a	779.8 ± 247.5b
163.136	n.a	n.i.	1.0 ± 1.0c	26.3 ± 18.3c	23 031.1 ± 10 269.4a	6287.6 ± 2163.1b
177.139	n.a	n.i.	0.0 ± 0.1c	0.8 ± 0.3c	2819.3 ± 1306.1a	665.3 ± 300.3b
225.267	n.a	n.i.	0 ± 0d	0.9 ± 0.2c	6033.2 ± 3921a	697.6 ± 169.7b

n.a., not available; n.i., not identified.

Mean ± standard deviation; values in a row with the same letter are not significantly different at 5%.

## CONCLUSION

The application of apigeninidin‐rich sorghum extract in maize dough fermented for 3 days led to the production of an orange‐red fermented dough with an enhanced antioxidant activity and stable production of sugars. However, phytate dephosphorylation and the production of VOCs in the dough were affected. Further investigations are recommended on (i) the characterisation of newly formed antioxidant compounds from enzymatic degradation of apigeninidin and (ii) the effect of apigeninidin on glycolytic enzyme activity and phytate dephosphorylation in relation to the applied fermentation process.

## Supporting information


**Figure S1**. Pictures of the dyed dough (A) and non‐dyed dough (B) at time 0 h.
