# Supplementary material for: Application of apigeninidin‐rich red sorghum biocolorant in a fermented food improves product quality
Source: J Sci Food Agric. 2018 Dec 4;99(4):2014–20. doi: 10.1002/jsfa.9427 (PMC6587490; doi:10.1002/jsfa.9427)
Supplement: Supplementary file 1 — Figure S1. Pictures of the dyed dough (A) and non‐dyed dough (B) at time 0 h. [file JSFA-99-2014-s001.rtf]

SUPPLEMENTARY INFORMATION
 
(A)	     			        (B)
Pictures of the dyed dough (A) and non-dyed dough (B) at time 0 h
